# The Association of Metastasis Pattern and Management of Metastatic Disease with Oncological Outcomes in Patients with Malignant Peripheral Nerve Sheath Tumors: A Multicenter Cohort Study

**DOI:** 10.3390/cancers13205115

**Published:** 2021-10-12

**Authors:** Ibtissam Acem, Enrico Martin, Winan J. van Houdt, Michiel A. J. van de Sande, Dirk J. Grünhagen, Cornelis Verhoef, MONACO Collaborators

**Affiliations:** 1 Department of Surgical Oncology and Gastrointestinal Surgery, Erasmus MC Cancer Institute, Dr. Molewaterplein 40, 3015 GD Rotterdam, The Netherlands; d.grunhagen@erasmusmc.nl (D.J.G.); c.verhoef@erasmusmc.nl (C.V.); 2 Department of Orthopedic Surgery, Leiden University Medical Center, Albinusdreef 2, 2333 ZA Leiden, The Netherlands; M.A.J.van_de_Sande@lumc.nl; 3 Department of Plastic and Reconstructive Surgery, University Medical Center Utrecht, Heidelberglaan 100, 3584 CX Utrecht, The Netherlands; E.Martin-2@umcutrecht.nl; 4 Department of Surgical Oncology, The Netherlands Cancer Institute, Plesmanlaan 121, 1066 CX Amsterdam, The Netherlands; w.v.houdt@nki.nl

**Keywords:** malignant peripheral nerve sheath tumor, distant metastasis, overall survival, prognostic factors, neurofibromatosis 1

## Abstract

**Simple Summary:**

Around 40% of patients with MPNSTs develop distant metastasis (DM) within five years. Identification of MPNST patients more likely to develop DM and the identification of prognostic factors after DM diagnosis may guide clinical decision-making and may result in a better balance between quantity and quality of life. This study aimed to identify clinicopathologic and treatment-related factors associated with the development of DM and with overall survival (OS) after DM diagnosis. NF1, high grade, tumor size, triton and R2 resections were independent risk factors for the development of DM. This is the first study that reveals that NF1 status is also independently associated with worse survival after DM diagnosis with a median survival difference of more than 6 months between NF1 and no-NF1 patients.

**Abstract:**

Purpose: This multicenter cohort study aimed to identify clinicopathologic and treatment-related factors associated with the development of distant metastasis (DM) and with overall survival (OS) after DM diagnosis in patients with malignant peripheral nerve sheath tumors (MPNST). Methods: All patients diagnosed with primary MPNST from 1988 to 2019 who were surgically treated for the primary tumor were included. Multivariable Cox regression analyses were performed to identify factors associated with DM and OS after DM diagnosis. Results: A total of 383 patients were included in this analysis, of which 150 developed metastatic disease. No differences in clinicopathologic characteristics and clinical outcome were found between patients with synchronous and metachronous DM. Neurofibromatosis type 1 (NF1), high grade, tumor size, triton and R2 resections were independent risk factors for the development of DM. NF1 and more than two metastasis sites were independently associated with worse OS after DM diagnosis. Metastasectomy, chemotherapy and the metastatic site category ‘other’ were associated with prolonged survival after DM diagnosis. Conclusions: This analysis provides important insights into clinicopathologic and treatment factors associated with outcomes in metastatic MPNST. Moreover, NF1-status is associated with a higher risk of DM; it is also independently associated with worse survival in metastatic MPNST.

## 1. Introduction

Approximately 30% of the patients with primary high-grade soft tissue sarcoma (STS) face metastatic disease within five years after primary treatment [1,2,3]. STS metastasize mainly to the lungs [4,5]. The median survival after distant metastasis (DM) is 1–2 years [4,6,7]. Metastatic disease is usually treated in a palliative setting. The mainstay treatment of metastatic STS is systemic therapy and metastasectomy for metachronous lung metastasis if the disease-free interval ≥ 1 year [8]. Especially in this setting, the right balance between life expectancy and quality of life is important. 

A better understanding of factors associated with metastatic disease and survival of metastatic disease may help to find a better balance between quantity and quality of life and enhance clinical decision-making. Several studies have assessed prognostic factors in metastatic STS [5,6,9,10,11,12,13,14]. However, studies on prognostic factors in metastatic malignant peripheral nerve sheath tumors (MPNSTs), a specific subtype of STS, are limited. 

In contrast to other STS subtypes, MPNSTs can originate within a (plexiform) neurofibroma, can occur in patients with neurofibromatosis type 1 (NF1) and can present with partial rhabdomyoblastic differentiation (triton tumor) [15,16]. In addition, the conventional three-level grading system, the FNCLCC grade, cannot be applied to MPNSTs due to its poor prognostic value [17]. 

Identification of MPNST patients more likely to develop DM and accurate prognosis after DM diagnosis may guide clinical decision-making and result in a better balance between quantity and quality of life. Therefore, we sought to characterize the impact of clinicopathologic and treatment characteristics on clinical outcomes in patients with metastatic MPNST treated in nine sarcoma centers in The Netherlands.

## 2. Materials and Methods

### 2.1. Patient Population

A retrospective cohort study of the nine Dutch sarcoma centers, the MONACO study, was undertaken after approval of the institutional review boards of the participating centers. All patients diagnosed with pathologically proven primary MPNST from 1988 to 2019 who were surgically treated for the primary tumor were included in this study. All patients were diagnosed in accordance with the World Health Organization classification of tumors of soft tissue and bone [18]. Patients with uncertain pathological reports or uncertain diagnosis based on available information during follow-up were excluded. In addition, patients with incorrectly registered time-to-event outcomes and patients who presented with local recurrence who were previously resected elsewhere were excluded.

### 2.2. Variables 

Patient, tumor and treatment characteristics and survival data were obtained from medical records. Age was determined as age at the time of diagnosis. The American Society of Anesthesiologist (ASA) classification system was used to categorize patients’ physical status [19]. Size was measured as the maximum diameter of tumor mass on imaging or based on pathology report. Tumor grade was categorized as low- and high-grade based on the Fédération Nationale des Centres de Lutte Contre le Cancer (FNCLCC) grading system. A tumor originating from below the investing fascia was categorized as deep-seated. A tumor was categorized as NF1-associated by confirmed genetic testing of an NF1 mutation or by clinical evaluation [20]. Surgical margin was categorized as R0 (microscopically negative), R1 (microscopically positive) or R2 (macroscopically positive). Tumor site was categorized as extremity, central (thorax, abdomen, pelvis, retroperitoneal), and head and neck. Triton status was extracted from pathological reports and was concluded either when stated as such in the report or when MPNST with rhabdomyoblastic differentiation was reported. Radiotherapy-associated MPNST was defined as previously delivered radiotherapy on the same site as the primary tumor bed. Metastatic sites were based on radiological reports. Metastatic site was categorized as pulmonary, extrapulmonary with or without pulmonary metastasis, and other. Extrapulmonary metastases were defined as liver, bone, brain and peritoneal metastasis. The ‘other’ category included lymph node metastasis and other rare metastatic sites. The number of metastatic sites was categorized as one site vs. two or more sites. The disease-free interval (DFI) was defined as the time between definitive surgery and the development of the first distant metastasis (DM) and was categorized as synchronous, ≤1 year and >1 year after definitive surgery. 

DM was defined as the first radiological or pathological evidence of recurrence at any other site outside the primary tumor bed. DM at presentation (synchronous metastasis) was defined as DM diagnosed within 3 months after date of diagnosis. DM developed after 3 months was categorized as metachronous metastasis. 

Endpoints of this study were DM and OS.

### 2.3. Statistical Analysis

All statistical analyses were performed in R (version 4.1.0) [21]. Baseline characteristics were described with proportions for categorical variables and means with standard deviations or medians with interquartile ranges (IQRs) for continuous variables. 

Overall survival (OS) was defined as the time interval between definitive surgery and death or date of last follow-up. Time-to-DM was defined as the time interval between definitive surgery and date of fist DM. Median survival was estimated with the reversed Kaplan–Meier estimator. Cumulative incidence of DM (CIDM) was estimated with death as the competing event. Differences in time-to-event outcomes were evaluated with the log-rank test. 

Multivariable Cox Proportional Hazards (PH) models were used to estimate the effect of several covariates on the development of metachronous DM and on OS after the first DM. The model for the development of DM included age, NF1, grade, tumor size, presence of triton, depth, tumor site, radiotherapy (RTX) for primary tumor, chemotherapy (CTX) for primary tumor and surgical margin. The model assessing the effect of different covariates on OS after first DM included age, NF1, size of primary tumor, grade, presence of triton, depth, number of metastatic sites, site of metastasis, DFI, metastasectomy and CTX for metastatic disease. 

Proportional hazards were assessed visually with the Schoenfeld residuals. 

Missing values were imputed using multiple imputations (MI) (m = 20), and estimates were pooled using Rubin’s rule [22]. 

A *p*-value ≤ 0.05 was considered statistically significant. Results from the Cox PH models were described in hazard ratios (HR) with 95% confidence intervals (CI). All statistical tests were two-sided. The packages ‘mice’ for MI, ‘survival’, ‘rms’ and ‘survminer’ were used for the survival and competing risk analyses. 

## 3. Results

A total of 481 patients were included in the MONACO study. Patients who presented with a local recurrence (*n* = 6), who were not treated surgically for the primary tumor (*n* = 64) and patients with incomplete time-to-event information (*n* = 28) were excluded in this analysis (Figure S1 Flow diagram). Of the 383 patients included in this study (Table S1 Baseline characteristics), 150 developed a DM during follow-up. The median follow-up was 47.9 months. The median follow-up in patients with metastatic MPNST was 23.7 months. Patient and tumor characteristics are summarized in Table 1. Thirty-six patients had a distant metastasis at presentation (9.40%). Fifty-seven patients (38.0%) had an MPNST in association with NF1. The median number of outpatient clinic visits of the total cohort after initial treatment was six times (IQR 3–6) in the first year, three times (IQR 3–4) in the second year, and three times (IQR 2–3) in the fourth and fifth year. 

Most of the patients with synchronous metastases had a metastasis at one site (80.6%). In addition, most of the patients with a first or second metachronous metastasis had the metastasis at one site (82.0% and 80.0%, respectively). Most metastases were located in the lung (66.7%, 75.6% and 63.3%, respectively) (Table 2). Synchronous metastases and first metachronous metastases were mainly treated with chemotherapy (53.3% and 37.6%, respectively) or surgery (30.0% and 28.2%, respectively) (Table 3). Most patients with second metachronous metastasis did not receive any treatment (33.3%). Doxorubicin monotherapy was the most-delivered first-line chemotherapy. 

### 3.1. Differences in Synchronous and First Metachronous Metastases

**Figure 1:**
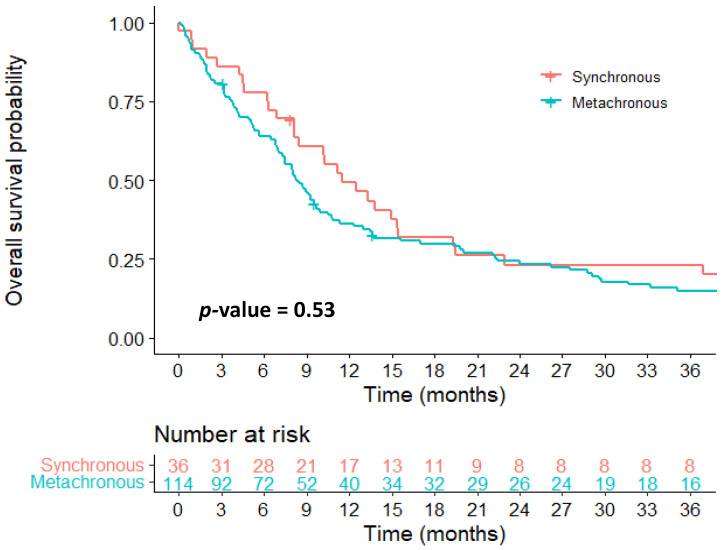
Survival plot of patients with synchronous vs. metachronous metastasis. *p*-value: Computed with log-rank test. Number at risk: Number of patients at risk of experiencing an event (death) at each time point (months) for synchronous and metachronous metastasis.

The incidence of synchronous DM was 9.40%. The incidence of metachronous DM was 30.5% at 5 years. As patients may develop both a synchronous and metachronous DM, the 5-year cumulative risk of a DM is 37.6%. MPNST patients with synchronous and first metachronous metastases were similar in respect to their baseline characteristics (Table S2). The median survival of patients with synchronous metastasis was 11.5 months (95%CI 8.11–19.3) compared with 8.28 months (95%CI 7.33–9.89) in patients with first metachronous metastasis (Figure 1). Patients diagnosed with a DM within 1 year and after 1 year after primary treatment had a median survival of 7.43 months (95%CI 4.90–9.50) and 9.89 (95%CI 7.95–19.8), respectively. 

### 3.2. Risk Factors for the Development of Metachronous Metastatic Disease in Primary MPNST

**Figure 2:**
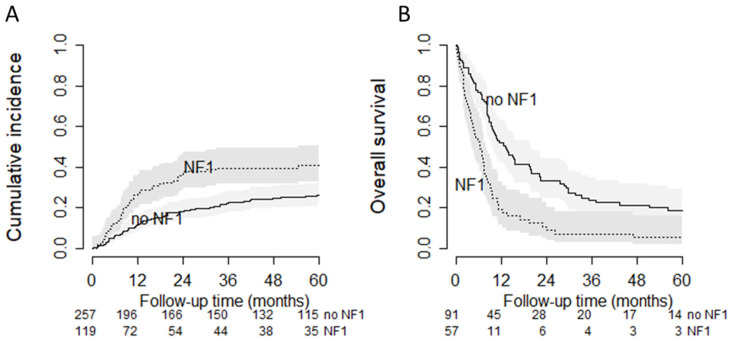
(**A**) Cumulative incidence of distant metastasis (**B**) and overall survival after distant metastasis stratified by neurofibromatosis-1 status.

**Figure 3:**
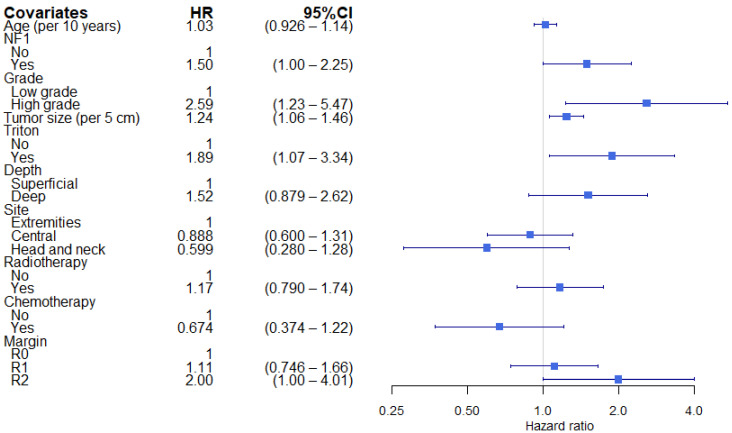
Multivariable cause-specific model for distant metastasis. Square represents the HR. End of horizontal line represents 95% CI. HR: hazard ratio, CI: confidence interval, NF1: neurofibromatosis type 1, cm: centimeter.

Patients with NF1-associated MPNST had a higher risk of developing DM. The 2-year CIDM in NF1 patients was 35.9% compared with 18.1% in no-NF1 patients (univariable HR 1.70; 95%CI 1.18–2.45) (Figure 2A). The increased risk of DM could only partially be explained by the imbalance in tumor and treatment characteristics in the multivariable cause-specific Cox model (HR 1.50; 95%CI 1.00–2.24) (Figure 3). Furthermore, high grade, tumor size, triton and R2 resections were independently associated with the development of DM.

### 3.3. Risk Factors for Overall Survival in Metastatic MNPST

**Figure 4:**
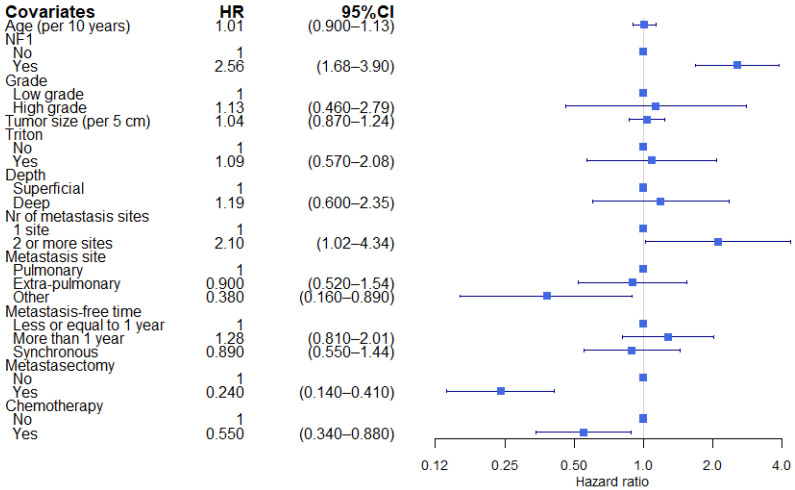
Multivariable Cox model on overall survival after first distant metastasis.Square represents the HR. End of horizontal line represents 95% CI. HR: hazard ratio, CI: confidence interval, NF1: neurofibromatosis type 1, cm: centimeter, Nr: number.

The median OS after metastatic MPNST was 8.9 months, with a 2-year OS of 23.9%. Patients with NF1-associated MNPST had a worse 2-year OS (10.5%) compared with no-NF1 patients (33.1%) (median OS: 6.31 and 13.0 months, respectively) (Table 1). The increased risk of mortality after DM in NF1 patients could not be explained by the imbalance of other tumor and treatment characteristics (HR 2.56; 95%CI 1.68–3.90) (Figure 4). Number of metastasis sites were also independently associated with a worse OS after DM diagnosis. The metastatic site category ‘other’, metastasectomy and chemotherapy for metastatic disease were independently associated with prolonged OS. Figure 2B depicts the overall survival of MPNST after the development of DM stratified by NF1. 

## 4. Discussion

The present study aimed to identify clinicopathologic and treatment-related factors associated with the development of DM and with OS after DM diagnosis. No differences in clinicopathologic characteristics and clinical outcomes were found between patients with synchronous and metachronous DM. NF1, high grade, tumor size, triton and R2 resections were independent risk factors for the development of DM. NF1 and more than two metastasis sites were independently associated with worse OS after DM diagnosis. Metastasectomy, chemotherapy and the metastatic site category ‘other’ were associated with better survival after DM diagnosis. 

### 4.1. Risk Factors for the Development of Metastatic Disease in Primary MPNST 

Consistent with the literature, this study demonstrated that size is an important prognostic factor for the development of DM in primary MPNST [23,24,25,26,27,28]. Site of the primary tumor and depth do not seem to be an independent risk factor for the development of DM [23,24,25,26,27,28]. However, literature review yields some contradictory results for the factors NF1, grade, triton and R2 resection. 

In Table 4, an overview of previous large (*n* > 100) cohort studies published after 2000 has been depicted. Seven out of eight studies assessed the effect of NF1 on DM. Five studies did not find a significant association between NF1 and DM. Some studies concluded that NF1-associated MPNST was not perse associated with worse outcome but had more adverse clinicopathological characteristics such as larger tumors, which might explain worse clinical outcomes [25,28]. However, the largest and most recent studies, including this study, revealed that NF1 is an independent risk factor for DM, independent of site, depth, grade, size and surgical margin [24]. The association between triton tumors and DM was only assessed in one other study [25]. In univariable analysis the association between triton and DM was significant, but in multivariable analysis, this association disappeared. Further studies are needed to better understand differences in tumor biology and clinical outcome in NF1-associated MPNST and triton tumors vs. sporadic MPNST and how this could be translated to optimal management of MPNST. Surgical margin was assessed in six studies. Studies in which surgical margin was categorized as positive vs. negative, no difference in DM risk was observed. However, studies in which the R classification was used, R2 resection was associated with higher risk of DM in uni- or multivariable analysis. Therefore, the R classification seems more informative than a dichotomous classification of surgical margin in MPNST. 

### 4.2. Risk Factors for Overall Survival in Metastatic MNPST

To the authors’ knowledge, this is the only study to date assessing prognostic factors for OS in synchronous and metachronous metastatic MPNST. One study assessed prognostic factors for OS in patients with synchronous metastasis only based on the SEER database [23]. However, this study was unable to assess the effect of DFI on OS and did not include MPNST specific information such as NF1-status. As only one study assessed OS after DM diagnosis in MPNST, we made an overview of previous large (*n* > 100) cohort studies assessing OS after DM diagnosis in all STS subtypes (Table S3). In accordance with most of the studies, size and depth of the primary tumor do not seem to be associated with OS after DM diagnosis [5,6,9,10,13,23,30,31,32,33]. However, the prognostic value of number of metastases or number of metastatic site and DFI has been subject of debate. Five studies, including this study, found an association between number of metastases or number of metastatic sites and worse OS after DM diagnosis, while six studies did not find an association [5,9,10,13,23,31,32,33,34,35]. Furthermore, the association between DFI and OS seems inconsistent between studies. Five studies did not find an association between the DFI and OS, while eight studies found a significant association [5,6,7,13,32,33,34,35,36,37]. Interestingly, five out of six studies of STS patients after pulmonary metastasectomy found a significant association between DFI and OS. It seems that the longer the DFI is, the better the OS after metachronous DM is [5,6,7,32,33,35,37]. This trend, although not significant, is also observed in our study. However, some studies showed worse OS in synchronous metastasis compared with metachronous metastasis, while others showed better OS in synchronous metastasis [5,34,37]. In our study, MPNST patients with synchronous metastasis do not seem to represent a more aggressive subgroup of tumors compared with patients who initially presented with nonmetastatic disease and experienced a DM at a later point in time. However, we only included patients with synchronous metastasis who received surgery for the primary tumor. Patients with synchronous metastasis who did not receive surgery for the primary tumor are likely to have poorer outcomes. 

Even though some older and smaller studies did not find an association between NF1 and OS, recent studies conclude that NF1 is associated with worse OS [24,38]. This multicenter study reveals that, besides the higher risk for DM, NF1 is also independently associated with worse OS after DM diagnosis. This might be explained by the higher risk of the development of second malignancies in MPNST patients with NF1 [39] or by a more aggressive tumor biology in NF1-associated metastatic MPNST. This underlines the potential added value of MPNST-specific information in prognostic tools and in clinical decision-making. 

### 4.3. Treatment of Metastatic MPNST

The optimal management of patients with metastatic MPNST is an important field of research. Palliative systemic therapy is the standard treatment in widespread metastatic disease [8]. However, metastasectomy is recommended in isolated resectable lung metastases (with a DFI ≥ 1 year), if complete excision of the lesions is feasible [8]. Especially in the metastatic setting, the anticipated side effects of these treatment modalities should be well balanced with the expected benefits. In our series, CTX, mainly monotherapy doxorubicin, was the most frequently offered treatment for synchronous and first metachronous disease followed by metastasectomy. However, the actual percentage of CTX in synchronous metastasis might be higher, as we only included patients who were surgically treated for the primary tumor. Patients with second metachronous metastasis mainly received best supportive care. 

Metastasectomy was the most important prognostic factor for better OS. The 2-year survival in patients with and without metastasectomy was 57.1% and 14.3%, respectively, in accordance with other studies [10,23,33,36,37]. Furthermore, this study found a significant association between systemic treatment and better OS in metastatic MPNST with a 2-year survival difference of 11.6% between patients with and without CTX. The improved survival after metastasectomy and CTX is most likely due to selection bias, as a selected group of patients with a generally overall better health status mainly receive these treatment options. Therefore, careful decision-making, taking all prognostic factors into consideration, is critical. 

### 4.4. Strengths and Limitations

This multicenter retrospective study has some inevitable limitations due to its retrospective design. Selective loss of follow-up and missing data might lead to selection bias. However, more than 90% of our study population was followed until death, and multiple imputation technique was used to reduce this risk of bias. Furthermore, no central review of pathology was performed. The diagnosis of MPNST can be challenging due to the lack of specific histologic criteria. A French cohort showed that after systematic review, 20% of the MPNSTs, mainly sporadic MPNSTs, were misclassified as MPNST [40]. Therefore, some MPNSTs might have been misclassified, which is an inherent limitation to all sarcoma studies without central pathology review. 

However, to our knowledge, this is the first nationwide study on metastatic MPNST to date including MPNST specific information. This design prevents selection bias and allows us to make inferences on the epidemiology of metastatic MPNST in an unselected patient population. As STS is a heterogeneous group of malignancies, research on single histological subtypes is vital to improve our understanding of tumor behavior, facilitate patient-tailored decision-making and find a right balance between quantity and quality of life. Unlike most population-based studies on (metastatic) MPNST, this study included important entity-specific information, such as NF1- and triton-status, and included clinicopathologic information on metachronous metastasis and follow-up. 

## 5. Conclusions

Almost 40% of the MPNST patients develop DM within five years. There are no differences in clinicopathological factors and oncological outcomes between synchronous and metachronous metastasis. High grade and R2 resections are mainly associated with the development of DM. Moreover, NF1-status is associated with a higher risk of DM; this is the first study that reveals that NF1-status is also independently associated with a worse survival in metastatic MPNST, with a median survival difference of more than 6 months. 

## Figures and Tables

**Table 1:** Baseline characteristics of 150 metastatic MPNST patients.

Variable	Overall (*n* = 150)	2-Year Survival after DM Diagnosis (95%CI)
Age (years)		
Median (IQR)	44 (29–59)	
Gender		
Female	69 (46.0%)	23.1 (14.9–35.8)
Male	81 (54.0%)	24.6 (16.7–36.3)
ASA		
I	70 (46.7%)	26.4 (17.8–39.1)
II	50 (33.3%)	19.9 (11.2–35.2)
III	7 (4.7%)	21.4 (4.20–100)
Missing	23 (15.3%)	
Tumor size (mm)		
Median (IQR)	70 (40–113)	
Missing	14 (9.3%)	
Depth		
Superficial	17 (11.3%)	45.8 (26.9–77.7)
Deep	124 (82.7%)	22.0 (15.8–30.9)
Missing	9 (6.0%)	
Grade		
Low grade	8 (5.3%)	37.5 (15.3–91.7)
High grade	141 (94.0%)	22.5 (16.5–30.8)
Missing	1 (0.7%)	
Site		
Extremities	70 (46.7%)	27.3 (18.5–40.3)
Central	70 (46.7%)	21.2 (13.4–33.6)
Head and neck	10 (6.7%)	20.0 (5.79–69.1)
NF1		
No	91 (60.7%)	33.1 (24.5–44.6)
Yes	57 (38.0%)	10.5 (4.94–22.4)
Missing	2 (1.3%)	
Neurofibroma		
Not in neurofibroma	130 (86.7%)	25.3 (18.8–34.2)
Within neurofibroma	18 (12.0%)	11.1 (3.01–41.0)
Missing	2 (1.3%)	
Triton		
No	133 (88.7%)	23.8 (17.6–32.3)
Yes	15 (10.0%)	19.6 (5.82–65.7)
Missing	2 (1.3%)	
RT-associated		
No	140 (93.3%)	25.0 (18.6–33.4)
Yes	9 (6.0%)	11.1 (1.75–70.5)
Missing	1 (0.7%)	
Site of metastasis		
Pulmonary only	89 (59.3%)	11.8 (4.83–29.1)
Extrapulmonary (±lung)	38 (25.3%)	24.6 (17.0–35.6)
Other	22 (14.7%)	38.1 (22.1–65.7)
Missing	1 (0.7%)	
Number of metastatic sites		
1 site	120 (80.0%)	25.8 (18.9–35.1)
2 or more sites	29 (19.3%)	13.8 (5.55–34.3)
Missing	1 (0.7%)	
Metastasectomy		
No	99 (66.0%)	14.3 (8.82–23.3)
Yes	39 (26.0%)	57.1 (43.2–75.6)
Missing	12 (8.0%)	
Chemotherapy		
No	80 (53.3%)	31.1 (22.3–43.4)
Yes	58 (38.7%)	19.5 (11.5–33.1)
Missing	12 (8.0%)	

**Table 2:** Metastasis pattern in MPNST.

Variable	Metastasis at Diagnosis (*n* = 36)	First Metachronous Metastasis (*n* = 123)	Second Metachronous Metastasis (*n* = 30)
Nr. of different metastasis sites			
1	29 (80.6%)	100 (82.0%)	23 (80.0%)
2	5 (13.9%)	18 (14.8%)	5 (13.3%)
>2	2 (5.56%)	4 (3.28%)	2 (6.67%)
Missing	0	3	0
Site			
Lung	24 (66.7%)	93 (75.6%)	19 (63.3%)
Liver	5 (13.9%)	9 (7.32%)	3 (10.0%)
Lymph node	5 (13.9%)	8 (6.50%)	5 (16.7%)
Bone	3 (8.33%)	17 (13.8%)	4 (13.3%)
Brain	1 (2.78%)	2 (1.63%)	2 (6.67%)
Peritoneal	5 (13.9%)	5 (4.07%)	2 (6.67%)
Other	3 (8.33%)	14 (11.4%)	4 (13.3%)
Missing	0	1	0

**Table 3:** Treatment pattern in metastatic MPNST.

Variable	Metastasis at Diagnosis (*n* = 36)	First Metachronous Metastasis (*n* = 123)	Second Metachronous Metastasis (*n* = 30)
Treatment of metastasis			
No treatment	5 (16.7%)	31 (26.5%)	10 (33.3%)
Metastasectomy	7 (23.3%)	26 (23.1%)	6 (20.0%)
Metastasectomy + RTX	-	4 (3.42%)	-
Metastasectomy + CTX	1 (3.33%)	1 (0.86%)	1 (3.33%)
Metastasectomy + RTX + CTX	1 (3.33%)	1 (0.86%)	2 (6.67%)
RTX	2 (6.67%)	11 (9.40%)	6 (20.0%)
CTX	12 (40.0%)	35 (29.9%)	4 (13.3%)
RTX + CTX	1 (3.33%)	7 (5.98%)	1 (3.33%)
RFA + CTX	1 (3.33%)	-	-
Missing	6	8	0
Treatment modality for metastasis			
No treatment	5 (16.7%)	31 (26.5%)	10 (33.3%)
Metastasectomy	9 (30.0%)	33 (28.2%)	9 (30.0%)
RTX	4 (13.3%)	23 (19.7%)	9 (30.0%)
CTX	16 (53.3%)	44 (37.6%)	8 (26.7%)
RFA	1 (3.33%)	-	-
Missing	6	8	0
First-line chemotherapy regimen			
Doxorubicin monotherapy	8 (50.0%)	13 (35.1%)	4 (50.0%)
Epirubicin monotherapy	1 (6.25%)	2 (5.41%)	-
Ifosfamide monotherapy	-	5 (13.5%)	1 (12.5%)
Doxorubicin + ifosfamide	3 (18.8%)	7 (18.9%)	2 (25.0%)
Epirubicin + ifosfamide	-	1 (2.70%)	-
Other	4 (25.0%)	9 (24.3%)	1 (12.5%)
Missing	0	7	0

**Table 4:** Overview of common predictors of DM in previous large (*n* > 100) cohort studies.

Study	*n*	Analysis	5-Year DMFS/5-Year DM-Rate	Factors Influencing Risk of DM ^a^						
				NF1	Site	Depth	Grade	Size	Triton	R2
Current study	383	MV	49.8/30.5	+	NS	NS	+	+	+	+
[23] «Xu, et al.» ^b^	764	MV	NR/NR	NA	NS	NA	NS *	+	NA	NA
[24] «Miao, et al.»	251	MV	60.6/NR	+	NS *	NS *	NS	+	NA	NS *
[25] «Watson, et al.» ^c^	225	MV	49.6/NR	NS	NS	NS *	NA	+	NS ^g,^*	NS ^d^
[29] «LaFemina, et al.»	105	UV	NR/NR	NS	NA	NA	NA	NA	NA	NA
[26] «Stucky, et al.» ^e^	175	UV	NR/NR	NS	NS	NS	+	+	NA	+
[27] «Zou, et al.»	113	MV	NR/37–69 ^f^	NS *	NS	NA	NA	+	NA	NS ^d^
[28] «Anghileri, et al.»	205	MV	NR/26.2	NS	NS	NA	+	+	NA	NS ^d^

*n*: number of patients, UV: univariable analyses, MV: multivariable analyses, DMFS: distant metastasis-free survival, DM-rate: distant metastasis rate, DM: distant metastasis, NF1: neurofibromatosis type 1, NR: not reported. ^a^ Significantly associated with lower DM risk (−), significantly associated with higher DM risk (+), not significantly associated (NS), not evaluated (NA). ^b^ Logistic regression on risk of DM at presentation. ^c^ High-grade MPNST. ^d^ Surgical margin defined as positive vs. negative. ^e^ Pearson’s chi-square/Fisher’s exact test used. ^f^ Five-year DM rate in patients with and without NF1 was 37% and 69%, respectively (death as competing risk not taken into account). ^g^ Sporadic MPNST vs. epithelioid type or triton tumor. * Significant in univariable analysis.

## Data Availability

The data that support the findings of this study are available on request from the corresponding author, upon reasonable request. The data are not publicly available due to information that could compromise the privacy of research participants.

## Supplementary Materials

The following are available online at www.mdpi.com/2072-6694/13/20/5115/s1, Figure S1: Consort flow diagram, Table S1: baseline characteristics of the total cohort of 383 patients with primary MPNST, Table S2: Baseline characteristics in patients with synchronous vs. metachronous metastasis, Table S3: Overview of common predictors of OS after DM diagnosis in previous large (*n* > 100) cohort studies.
